# 

**Published:** 1909-06

**Authors:** 


### CONTENTS—JUNE, 1909.—VOL. XXIV, NO. 12.

ORIGINAL ARTICLES—

Acute Traumatic Tetanus Treated by Magnesium Sulphate, With Report of a Case in the Treatment of Which Injections of an Aqueous Twenty-five Per Cent Solution of Magnesium Sulphate Were Made in the Spinal Subarachnoid Space; With Recovery. By Aime Paul Heineck, Chicago, Ill. .......... 499

EDITORIAL—

The Passing of Aristocracy in Medicine .......... 515
The Indomitable Spirit .......... 514

CONTRIBUTED—

Motherhood .......... 521
Sympathy .......... 521

ABSTRACTS AND SELECTIONS .......... 522

PUBLISHER'S DEPARTMENT .......... 531

